# Supplementary material for: A QCT View of the Interplay between Hydrogen Bonds and Aromaticity in Small CHON Derivatives
Source: Molecules. 2022 Sep 16;27(18):6039. doi: 10.3390/molecules27186039 (PMC9504421; doi:10.3390/molecules27186039)
Supplement: Supplementary file 1 [file molecules-27-06039-s001.zip › molecules-1879616-SI.pdf]

Supporting Information

A QCT view of the interplay between hydrogen bonds and aromaticity in small CHON derivatives

Miguel Gallegos, Daniel Barrena-Espés, José Manuel Guevara-Vela,
Tomás Rocha-Rinza, Ángel Martín Pendás.

Contents

1 Aromaticity Indexes 3

2 Electronic energies 4

3 IQA energetic partitioning 4

4 Group energies 7

5 QTAIM descriptors 8

6 Optimised geometries 10

7 Other AZH (DCR) isomers 16

7.1 Optimised geometries . . . 16

7.2 Energies and aromaticity metrics . . . 17

7.3 QTAIM and IQA values . . . 17

1 Aromaticity Indexes

FLU index

The FLU aromaticity index for a ring of n atoms and with a connectivity given by $\mathcal{A} = (A_1, A_2, ..., A_n)$ is computed as:

$$\mathrm{FLU}(\mathcal{A}) = \frac{1}{n}\sum_{i=1}^{n}\left[\left(\frac{\delta(A_i)}{\delta(A_{i-1})}\right)^{\alpha}\left(\frac{\delta(A_i, A_{i-1}) - \delta_{\mathrm{ref}}(A_i, A_{i-1})}{\delta_{\mathrm{ref}}(A_i, A_{i-1})}\right)\right]^2, \tag{1}$$

where the summation runs over all adjacent atomic pairs A_i–A_{i-1} in the ring, $\delta(A_i)$ is the atomic delocalisation index of atom A_i, $\delta(A_i, A_{i-1})$ is the Electron Sharing Index (ESI) for atomic pair A_i–A_{i-1} in the system while $\delta_{\mathrm{ref}}(A_i, A_{i-1})$ is the ESI for that pair in the aromatic ring chosen as reference. The α exponent is used to ensure that the atomic valence ratio (first term within the summation) is greater than one by taking the values as follows:

$$\alpha = \begin{cases} 1 & \delta(A_i) > \delta(A_{i-1}) \\ -1 & \delta(A_i) \leq \delta(A_{i-1}) \end{cases} \tag{2}$$

MCI index

The MCI index is computed according to the following expression:

$$\mathrm{MCI}(\mathcal{A}) = \frac{1}{2n}\sum_{\mathcal{P}(\mathcal{A})} I_{\mathrm{ring}}(\mathcal{A}), \tag{3}$$

where $\mathcal{P}(\mathcal{A})$ accounts for the $n!$ possible permutations of the atomic distribution $\mathcal{A}$, and I_{ring} is the multicenter index bonding whose expression is given by:

$$I_{ring} = 2^{n-1}\sum_{i_1 i_2 \ldots i_n} S_{i_1,i_2}(A_1) \ldots S_{i_n,i_1}(A_n), \tag{4}$$

where $S_{ij}(A_1)$ is the atomic overlap matrix of atom A_1,

$$S_{ij}(A_1) = \int_{A_1} \phi_i^*(\mathbf{1})\phi_j(\mathbf{1})d\mathbf{1}, \tag{5}$$

wherein $\phi_i(\mathbf{1})$ is a natural orbital.

Table S1 gathers the absolute values of the aromaticity metrics for the studied monomers and dimers.

Table S1: MCI and FLU indexes for the ACR and DCR tautomers of the monomers and dimers examined in the paper along with its change upon formation of the corresponding molecular clusters.

System	$\mathrm{MCI}_{\mathrm{mono}}$	$\mathrm{MCI}_{\mathrm{dimer}}$	$\mathrm{FLU}_{\mathrm{mono}}$	$\mathrm{FLU}_{\mathrm{dimer}}$	ΔMCI	ΔFLU
AZH (ACR)	−0.007	−0.006	0.084	0.067	0.001	−0.017
2HP (ACR)	0.054	0.046	0.004	0.005	−0.008	0.001
AZA (ACR)	−0.008	−0.004	0.066	0.045	0.004	−0.021
2AP (ACR)	0.049	0.044	0.005	0.006	−0.004	0.001
AZH (DCR)	0.010	0.009	0.068	0.063	−0.001	−0.005
2HP (DCR)	0.018	0.025	0.030	0.020	0.007	−0.010
AZA (DCR)	0.010	0.009	0.061	0.054	−0.001	−0.007
2AP (DCR)	0.015	0.022	0.029	0.019	0.007	−0.010

2 Electronic energies

This section reports the electronic energies of the monomers and dimers, computed at the DFT and CC levels of theory.

Table S2: Electronic energies of the ACR and DCR monomers and dimers considered in this paper. Atomic units are used throughout.

ACR system	$E^{\text{DFT}}_{\text{mono}}$	$E^{\text{DFT}}_{\text{dimer}}$	$E^{\text{CC}}_{\text{mono}}$	$E^{\text{CC}}_{\text{dimer}}$
AZH (ACR)	−245.789875	−491.640735	−245.676321	−491.405934
NCO (ACR)	−169.749941	−339.556373	−169.686727	−339.421419
2HP (ACR)	−323.275034	−646.598502	−323.101812	−646.246795
AZA (ACR)	−225.939556	−451.921827	−225.815372	−451.667753
2AP (ACR)	−303.418926	−606.859407	−303.236530	−606.490687
NCN	−149.895441	−299.817573	−149.822472	−299.666681
AZH (DCR)	−245.853376	−491.721935	−245.737398	−491.487883
NCO (DCR)	−169.779384	−339.584147	−169.713194	−339.448050
2HP (DCR)	−323.283894	−646.604505	−323.108275	−646.249409
AZA (DCR)	−225.964891	−451.953075	−225.841051	−451.702424
2AP (DCR)	−303.397236	−606.834174	−303.214606	−606.462894

Table S3: Electronic binding energies, calculated with the DFT and CC approximations, for the dimerisation of the ACR tautomers. The values are reported in kcal/mol.

ACR system	$E^{\text{DFT}}_{\text{bind}}$	$E^{\text{CC}}_{\text{bind}}$
AZH	−38.27	−33.44
NCO	−35.45	−30.10
2HP	−30.39	−27.09
AZA	−26.80	−23.22
NCN	−16.75	−13.64
2AP	−13.53	−11.06

Table S4: Electronic binding energies, calculated with the DFT and CC approximations, for the dimerisation of the DCR tautomers. The values are reported in kcal/mol.

DCR system	$E^{\text{DFT}}_{\text{bind}}$	$E^{\text{CC}}_{\text{bind}}$
AZH	−9.53	−8.21
NCO	−15.93	−13.59
2HP	−23.04	−20.62
AZA	−14.62	−12.75
NCN	−16.75	−13.64
2AP	−24.91	−21.14

3 IQA energetic partitioning

This section reports the absolute values of the energetic terms resulting from the IQA energetic decomposition of the monomers and dimers investigated in the paper.

Table S5: Delocalisation indices (DI) and IQA interaction energies (E_{int}) along with their exchange-correlation (E_{xc}) and electrostatic components (E_{cl}) for the main atoms involved in the dimerisation process investigated herein. All energies are given in Hartrees whereas the DI are given in electrons. The labeling of the atoms in shown in Figure 3 in the main body of the paper.

System	D−H				H···A				D−C				C−A			
Dimers	DI	E_{int}	E_{xc}	E_{cl}	DI	E_{int}	E_{xc}	E_{cl}	DI	E_{int}	E_{xc}	E_{cl}	DI	E_{int}	E_{xc}	E_{cl}
AZH (ACR)	0.391	-0.565	-0.132	-0.433	0.134	-0.267	-0.035	-0.232	0.998	-1.229	-0.313	-0.916	1.318	-0.994	-0.409	-0.584
NCO (ACR)	0.390	-0.490	-0.125	-0.366	0.197	-0.314	-0.053	-0.261	0.988	-1.195	-0.309	-0.886	1.387	-1.241	-0.418	-0.824
2HP (ACR)	0.428	-0.522	-0.138	-0.384	0.152	-0.285	-0.040	-0.246	0.932	-1.043	-0.296	-0.748	1.165	-0.985	-0.366	-0.619
AZA (ACR)	0.514	-0.482	-0.163	-0.319	0.165	-0.231	-0.042	-0.189	1.218	-1.137	-0.374	-0.763	1.234	-0.828	-0.386	-0.442
2AP (ACR)	0.634	-0.474	-0.203	-0.272	0.103	-0.185	-0.023	-0.162	1.072	-0.912	-0.339	-0.573	1.182	-0.879	-0.370	-0.509
NCN	0.609	-0.478	-0.194	-0.284	0.117	-0.197	-0.027	-0.170	1.109	-1.030	-0.345	-0.685	1.451	-1.111	-0.435	-0.676
AZH (DCR)	0.708	-0.421	-0.224	-0.197	0.066	-0.141	-0.014	-0.127	0.910	-0.777	-0.285	-0.492	1.306	-1.468	-0.399	-1.069
NCO (DCR)	0.626	-0.480	-0.200	-0.280	0.099	-0.188	-0.023	-0.165	1.096	-1.169	-0.340	-0.829	1.217	-1.425	-0.375	-1.050
2HP (DCR)	0.558	-0.491	-0.181	-0.311	0.132	-0.224	-0.033	-0.191	0.979	-1.012	-0.309	-0.702	1.135	-1.292	-0.358	-0.934
AZA (DCR)	0.609	-0.471	-0.196	-0.274	0.099	-0.191	-0.022	-0.169	0.955	-0.838	-0.303	-0.535	1.495	-1.203	-0.446	-0.757
2AP (DCR)	0.529	-0.485	-0.171	-0.315	0.163	-0.235	-0.041	-0.195	1.001	-0.904	-0.316	-0.588	1.358	-0.968	-0.418	-0.550
Monomers																
AZH (ACR)	0.607	-0.540	-0.188	-0.352					0.919	-1.061	-0.287	-0.774	1.453	-1.029	-0.445	-0.585
NCO (ACR)	0.635	-0.528	-0.196	-0.332					0.888	-0.996	-0.275	-0.721	1.558	-1.252	-0.460	-0.791
2HP (ACR)	0.635	-0.524	-0.197	-0.328					0.876	-0.909	-0.275	-0.634	1.229	-0.947	-0.385	-0.562
AZA (ACR)	0.776	-0.458	-0.240	-0.219					1.086	-1.041	-0.339	-0.702	1.401	-0.882	-0.432	-0.450
2AP (ACR)	0.785	-0.452	-0.243	-0.209					1.021	-0.874	-0.324	-0.550	1.217	-0.871	-0.380	-0.490
NCN	0.790	-0.450	-0.244	-0.206					1.035	-0.974	-0.324	-0.650	1.527	-1.135	-0.453	-0.682
AZH (DCR)	0.810	-0.400	-0.248	-0.152					0.876	-0.747	-0.272	-0.475	1.349	-1.490	-0.411	-1.079
NCO (DCR)	0.788	-0.450	-0.243	-0.207					1.027	-1.102	-0.320	-0.782	1.287	-1.465	-0.394	-1.071
2HP (DCR)	0.767	-0.456	-0.239	-0.217					0.913	-0.958	-0.289	-0.670	1.226	-1.370	-0.383	-0.986
AZA (DCR)	0.761	-0.447	-0.238	-0.209					0.912	-0.820	-0.289	-0.531	1.548	-1.209	-0.459	-0.750
2AP (DCR)	0.763	-0.460	-0.239	-0.221					0.934	-0.862	-0.296	-0.566	1.464	-1.006	-0.445	-0.561

Table S6: Change in the DI and IQA interaction energies upon dimerisation. All energies are given in Hartrees whereas the DI are given in electrons.

System	D−H				H···A			D−C				C−A			
	ΔDI	ΔE_{int}	ΔE_{xc}	ΔE_{cl}	E_{int}	E_{xc}	E_{cl}	ΔDI	ΔE_{int}	ΔE_{xc}	ΔE_{cl}	ΔDI	ΔE_{int}	ΔE_{xc}	ΔE_{cl}
AZH (ACR)	−0.216	−15.94	34.76	−50.70	−167.54	−21.90	−145.64	0.079	−105.11	−16.06	−89.04	−0.136	22.53	22.22	0.31
NCO (ACR)	−0.246	23.66	44.99	−21.34	−197.10	−33.45	−163.65	0.100	−124.87	−21.40	−103.48	−0.171	6.59	26.80	−20.21
2HP (ACR)	−0.207	1.57	36.52	−34.95	−178.97	−24.79	−154.18	0.056	−84.27	−13.11	−71.16	−0.064	−23.91	11.99	−35.90
AZA (ACR)	−0.262	−14.85	47.79	−62.65	−144.92	−26.38	−118.54	0.131	−60.01	−21.77	−38.23	−0.167	33.44	28.54	4.90
2AP (ACR)	−0.151	−13.87	25.46	−39.32	−116.07	−14.63	−101.44	0.051	−24.08	−9.59	−14.48	−0.034	−5.27	6.25	−11.52
NCN	−0.181	−17.94	31.13	−49.07	−123.57	−16.86	−106.71	0.073	−34.95	−13.20	−21.75	−0.076	15.30	11.72	3.58
AZH (DCR)	−0.103	−12.87	15.13	−28.00	−88.66	−8.67	−79.99	0.034	−18.43	−7.93	−10.50	−0.043	13.48	7.52	5.95
NCO (DCR)	−0.162	−18.79	26.86	−45.66	−118.00	−14.30	−103.70	0.069	−41.94	−12.42	−29.52	−0.070	25.52	11.96	13.56
2HP (DCR)	−0.209	−22.09	36.58	−58.67	−140.41	−20.67	−119.74	0.065	−33.52	−12.91	−20.60	−0.091	48.69	16.08	32.61
AZA (DCR)	−0.152	−15.00	25.98	−40.98	−119.73	−13.87	−105.86	0.043	−11.55	−9.04	−2.51	−0.053	4.02	8.60	−4.58
2AP (DCR)	−0.234	−16.00	42.67	−58.67	−147.72	−25.54	−122.18	0.067	−26.29	−12.86	−13.43	−0.106	23.91	17.01	6.90

Figures S1 and S2 show the change in the IQA energetic components of the most relevant interactions upon the dimerisation of the systems shown in Figure 1 in the main body of the paper.

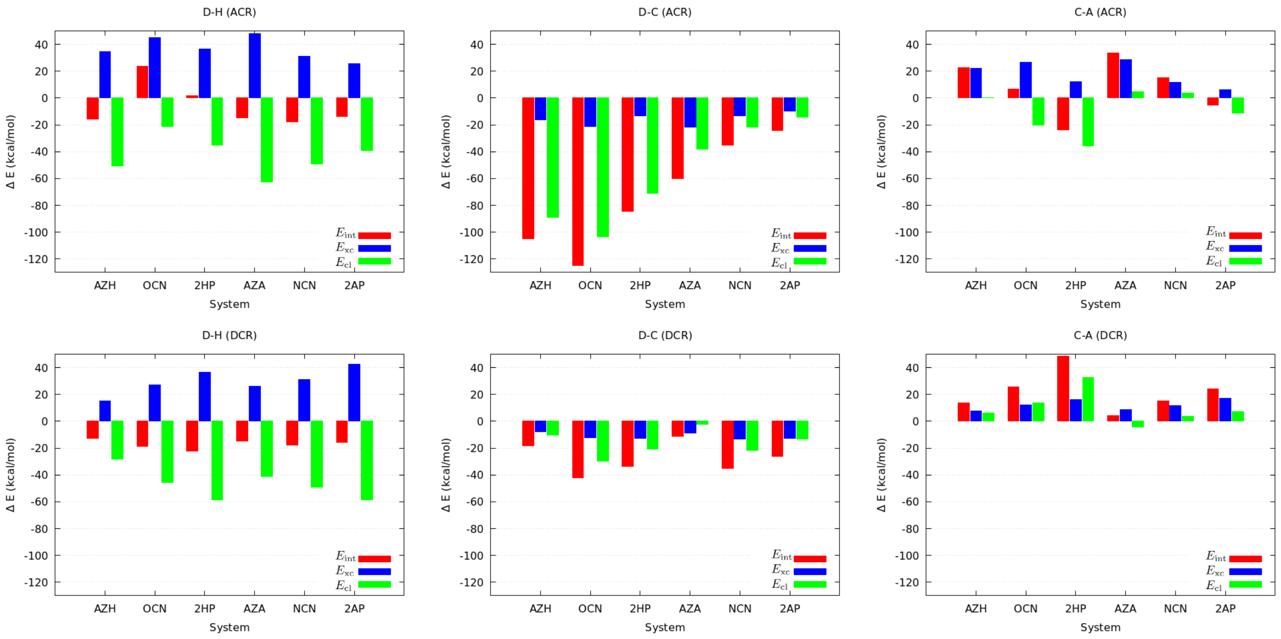

Figure S1: Change in the IQA interaction energies upon dimerisation.

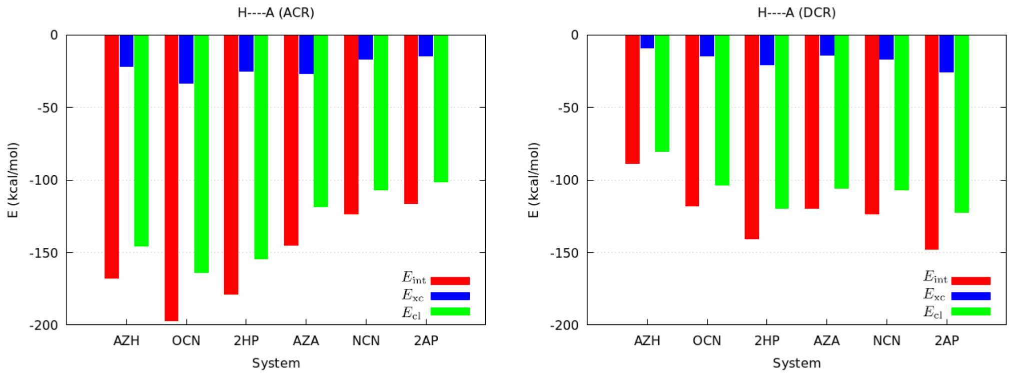

Figure S2: IQA interaction energies, along with its constituting components, for the H···A interatomic pair of the dimers investigated in the paper.

4 Group energies

The IQA partitioning splits the electronic energy as a sum of one- and two-body contributions. Thus, under the IQA theoretical framework, group energies become readily available by summing the self energies of all the the constituting atoms along with all their pairwise interaction energies, as:

$$E^G = \sum_A^N E_{\text{self}}^A + \sum_A^N \sum_{A>B}^N E_{\text{int}}^{AB}, \tag{6}$$

for a chemical fragment (G) formed by N atoms. Following this approach, the group energies of the whole monomers along with their constituting rings were obtained. For the latter, only the atoms embedded in the cyclic structure (without any of the decorating substituent) were considered.

Table S7 collects the decomposition of the binding energies, as estimated by the IQA partition, in terms of the self energy of each monomer along with the interaction between them.

Table S7: Change in the IQA group energies upon the dimerisation process. The difference in between the self (E_{self}) and interaction (E_{int}) energies (ΔE) is computed as the sum of twice the self energy plus the interaction energy ($\Delta E = 2 * E_{\text{self}} + E_{\text{int}}$). All values are reported relative to their reference system and are given in kcal/mol.

System	ΔE_{self}	ΔE_{int}	ΔE
AZH (ACR)	−14.85	+27.11	−2.58
2HP (ACR)	−14.22	+34.55	+6.11
AZA (ACR)	+17.84	−45.07	−9.39
2AP (ACR)	−4.15	+12.43	+4.12
AZH (DCR)	−9.43	+25.65	+6.78
2HP (DCR)	+12.07	−30.26	−6.12
AZA (DCR)	−4.81	+11.94	+2.32
2AP (DCR)	+15.18	−37.26	−6.91

Given the uncertainty inherent to the numerical integration employed for the energetic partitioning, the IQA binding energies are slightly offset (by roughly 1 kcal/mol) with respect to the DFT values.

Table S8 gathers the change in the IQA energies of the 6 or 4 membered rings upon dimerisation.

Table S8: Change in the IQA ring energies (along with their intra-atomic and interaction components) upon the dimerisation process. All values are given in kcal/mol.

System	ΔE_{ring}	ΔE_{intra}	ΔE_{int}
AZH (ACR)	48.8	35.8	13.0
2HP (ACR)	38.0	56.2	−18.2
AZA (ACR)	34.7	23.1	11.6
2AP (ACR)	15.0	20.0	−5.0
AZH (DCR)	−16.5	4.4	−21.0
2HP (DCR)	−42.6	9.0	−51.6
AZA (DCR)	−8.9	3.8	−12.8
2AP (DCR)	−23.7	20.6	−44.3

5 QTAIM descriptors

This section reports selected QTAIM descriptors for the monomers and dimers of the studied systems along with a brief introduction to the concept of delocalisation index (DI) in the QTAIM realm.

The Quantum Theory of Atoms in Molecules (QTAIM) is a real space tool formulated within the field of Quantum Chemical Topology (QCT), which divides the whole space in a collection of non-overlapping and well defined basins (Ω) corresponding to the atomic domains of a chemical system. Starting from these, it is then possible to obtain a wide variety of chemically intuitive and physically rigorous local and global electronic descriptors form the integration of the adequate quantum mechanical operators. Within the former, and in the context of chemical bonding, the delocalisation index (DI) becomes particularly useful, providing an estimation of the average electron delocalisation between any two entities. More specifically, the delocalisation index between any two atoms A and B, δ(A,B), can be obtained though the integration of the XC density (ρ_{XC}) within the corresponding QTAIM basins, as given by:

$$\delta(A,B) = 2\int_{\Omega_A}\int_{\Omega_B} \rho_{XC}(r_1,r_2)dr_1dr_2. \quad (7)$$

And thus, it provides an estimation of the average number of electrons shared between the aforementioned basins, being hence a measure of the covariance of the electron populations of those basins and thus, their delocalisation.

Table S9: QTAIM atomic charges of the main atoms involved in the HB interactions established upon dimerisation. All values are given in electrons. The atom labeling is shown in Figure 3 in the main body of the paper.

System	Dimers				Monomers			
	Q(D)	Q(H)	Q(A)	Q(C)	Q(D)	Q(H)	Q(A)	Q(C)
AZH (ACR)	-1.248	0.683	-1.120	1.355	-1.159	0.618	-1.044	1.310
NCO (ACR)	-1.223	0.637	-1.280	1.429	-1.139	0.596	-1.208	1.344
2HP (ACR)	-1.220	0.640	-1.266	1.196	-1.134	0.591	-1.199	1.127
AZA (ACR)	-1.320	0.557	-1.099	1.147	-1.259	0.444	-1.036	1.149
2AP (ACR)	-1.260	0.504	-1.230	1.029	-1.220	0.434	-1.208	1.009
NCN	-1.283	0.517	-1.239	1.224	-1.232	0.429	-1.216	1.216
AZH (DCR)	-1.059	0.454	-1.205	1.414	-1.022	0.390	-1.181	1.425
NCO (DCR)	-1.282	0.519	-1.227	1.536	-1.230	0.433	-1.203	1.543
2HP (DCR)	-1.300	0.543	-1.240	1.365	-1.231	0.438	-1.217	1.405
AZA (DCR)	-1.224	0.526	-1.253	1.181	-1.194	0.452	-1.219	1.188
2AP (DCR)	-1.307	0.540	-1.218	1.068	-1.244	0.439	-1.187	1.080

Figure S3 shows the correlation of the DFT binding energies and the electron density at the bond critical point (BCP) between the HB contacts formed upon dimerisation.

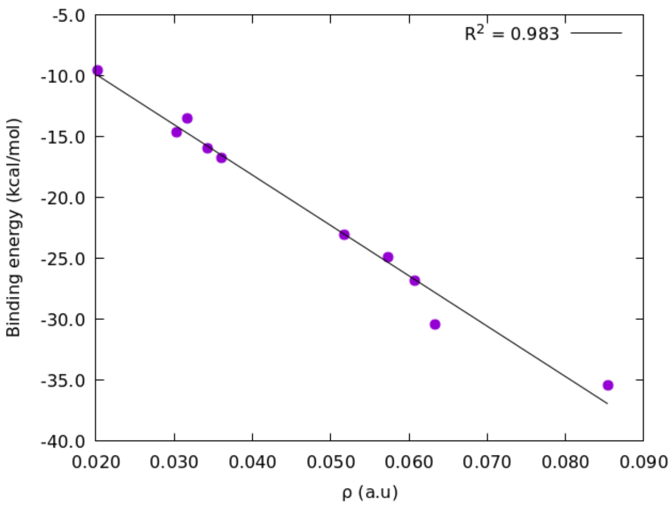

Figure S3: Correlation of the DFT dimerisation energy as a function of the electron density at the BCP of the intermolecular HB contacts. Only the bona fide local minima (optimised without any constraint) are shown.

6 Optimised geometries

The current section reports the optimised geometries, reported in Å as Cartesian coordinates, for the systems under study.

Table S10: 2AP (ACR): 2-aminopyridine dimer

Atom	x	y	z
N	−0.508650	1.796479	0.000246
N	0.508651	−1.796480	0.000246
N	1.621136	0.944142	0.000444
N	−1.621140	−0.944140	0.000444
C	−0.919010	4.156858	−0.000300
C	0.919013	−4.156860	−0.000300
C	0.811952	2.021264	0.000188
C	−0.811950	−2.021270	0.000188
C	−1.331900	2.841135	0.000008
C	1.331896	−2.841130	0.000009
C	0.454950	4.392173	−0.000370
C	−0.454950	−4.392170	−0.000370
C	1.328530	3.333330	−0.000120
C	−1.328530	−3.333330	−0.000120
H	−1.637730	4.965039	−0.000490
H	1.637727	−4.965040	−0.000490
H	−2.392580	2.601625	0.000073
H	2.392577	−2.601620	0.000073
H	0.838241	5.406695	−0.000610
H	−0.838240	−5.406700	−0.000610
H	2.400856	3.491217	−0.000160
H	−2.400860	−3.491220	−0.000160
H	1.232259	−0.003800	0.000677
H	2.614012	1.073115	0.000412
H	−2.614010	−1.073120	0.000412
H	−1.232260	0.003798	0.000677

Table S11: 2AP (DCR): 2-aminopyridine dimer.

Atom	x	y	z
N	−0.527512	1.739915	0.000002
N	0.500697	−1.734462	0.000002
N	1.565159	0.799194	0.000003
N	−1.592551	−0.794231	0.000002
C	−0.956829	4.065624	−0.000002
C	0.930137	−4.060296	−0.000002
C	0.839649	1.878851	0.000001
C	−0.866377	−1.873596	0.000001
C	−1.382665	2.773557	0.000000
C	1.355975	−2.768251	0.000000
C	0.440410	4.281216	−0.000002
C	−0.467130	−4.276066	−0.000002
C	1.309502	3.236639	0.000000
C	−1.336241	−3.231534	−0.000001
H	−1.663199	4.882437	−0.000003
H	1.636664	−4.877020	−0.000003
H	−2.431348	2.500624	0.000001
H	2.404595	−2.495121	0.000001
H	0.825714	5.295288	−0.000003
H	−0.852228	−5.290228	−0.000003
H	2.380440	3.399800	0.000000
H	−2.407237	−3.394310	−0.000001
H	0.903851	−0.749996	0.000002
H	2.550298	1.030778	0.000002
H	−2.577384	−1.027092	0.000001
H	−0.929543	0.755040	0.000003

Table S12: 2HP (DCR): 2-hydroxypyridine dimer.

Atom	x	y	z
O	−1.521370	0.882220	0.000004
O	1.521371	−0.882220	0.000003
N	0.610136	1.669091	0.000001
N	−0.610140	−1.669090	0.000001
C	−1.173080	3.226355	0.000001
C	1.173081	−3.226360	0.000001
C	−0.260990	4.237915	−0.000001
C	0.260991	−4.237920	−0.000001
C	1.123880	3.970401	−0.000002
C	−1.123880	−3.970400	−0.000002
C	1.512505	2.667017	−0.000001
C	−1.512510	−2.667020	−0.000001
C	−0.753190	1.857792	0.000002
C	0.753193	−1.857790	0.000002
H	−0.604940	5.266838	−0.000002
H	0.604934	−5.266840	−0.000002
H	−2.238770	3.411889	0.000002
H	2.238769	−3.411890	0.000002
H	1.857988	4.762805	−0.000004
H	−1.857990	−4.762800	−0.000004
H	0.949772	0.678709	0.000002
H	−0.949770	−0.678710	0.000002
H	2.549822	2.356066	−0.000002
H	−2.549820	−2.356060	−0.000001

Table S13: 2HP (ACR): 2-hydroxypyridine dimer.

Atom	x	y	z
O	−1.622211	0.875389	0.000001
O	1.620940	−0.873710	0.000001
N	0.557055	1.561239	−0.000002
N	−0.558440	−1.559414	−0.000002
C	−1.197008	3.180308	0.000002
C	1.195568	−3.178528	0.000002
C	−0.268587	4.192937	0.000001
C	0.267127	−4.191116	0.000001
C	1.092217	3.887119	0.000000
C	−1.093664	−3.885280	−0.000001
C	1.446023	2.557759	−0.000002
C	−1.447426	−2.555912	−0.000002
C	−0.742973	1.853242	0.000000
C	0.741595	−1.851437	0.000000
H	−0.597131	5.226201	0.000002
H	0.595636	−5.224391	0.000002
H	−2.262022	3.369828	0.000003
H	2.260573	−3.368102	0.000003
H	1.849228	4.659460	−0.000001
H	−1.850668	−4.657623	−0.000001
H	1.159444	0.032816	−0.000001
H	−1.160388	−0.030940	−0.000001
H	2.488905	2.255477	−0.000003
H	−2.490282	−2.253538	−0.000003

Table S14: AZA (ACR): azet-2-amine dimer.

Atom	x	y	z
N	−0.670630	−1.612360	−0.000001
N	0.670626	1.612361	−0.000001
N	1.613950	−0.917570	0.000001
N	−1.613950	0.917566	0.000001
C	0.660925	−1.795850	0.000000
C	−0.660930	1.795853	0.000000
C	0.592075	−3.303220	0.000000
C	−0.592080	3.303219	0.000000
C	−0.731180	−3.055520	−0.000001
C	0.731182	3.055517	−0.000001
H	−1.635400	−3.653960	−0.000003
H	1.635404	3.653961	−0.000003
H	1.264629	−4.143080	0.000000
H	−1.264630	4.143084	0.000000
H	−1.376810	−0.127380	0.000001
H	1.376813	0.127382	0.000001
H	−2.566290	1.240207	0.000003
H	2.566288	−1.240210	0.000002

Table S15: AZA (DCR): azet-2-amine dimer.

Atom	x	y	z
N	−0.533564	−1.634761	0.000005
N	0.542036	1.659243	0.000003
N	1.781986	−0.991012	0.000008
N	−1.773294	1.015217	0.000003
C	0.855767	−1.844284	0.000004
C	−0.847337	1.868727	0.000001
C	0.623086	−3.331521	−0.000008
C	−0.614674	3.355964	−0.000005
C	−0.692756	−3.027361	0.000003
C	0.701182	3.051794	0.000000
H	−1.628855	−3.568629	0.000003
H	1.637148	3.593290	0.000000
H	1.231450	−4.216550	−0.000019
H	−1.222818	4.241144	−0.000012
H	−1.128073	−0.803865	0.000005
H	1.135897	0.828056	0.000004
H	−2.683306	1.465289	0.000000
H	2.691726	−1.441652	0.000003

Table S16: AZH (DCR): azet-2-ol dimer.

Atom	x	y	z
O	1.136533	−1.539830	−0.203310
O	−1.136530	1.539824	−0.203310
N	1.109523	1.367361	0.623121
N	−1.109520	−1.367360	0.623123
C	−0.028850	1.957031	−0.059250
C	0.028849	−1.957030	−0.059250
C	0.806930	3.117438	−0.484940
C	−0.806930	−3.117440	−0.484940
C	1.842760	2.455377	0.055933
C	−1.842760	−2.455380	0.055933
H	2.910030	2.607292	0.143615
H	−2.910030	−2.607290	0.143613
H	0.635300	4.074784	−0.945250
H	−0.635300	−4.074780	−0.945250
H	−1.351590	−0.403590	0.386784
H	1.351596	0.403585	0.386785

Table S17: AZH (ACR): azet-2-ol dimer.

Atom	x	y	z
O	0.987733	1.364924	−0.142983
O	−1.249874	−1.717334	−0.071040
N	1.241556	−0.975714	−0.149604
N	−1.502153	0.623178	−0.048121
C	−3.392474	−0.321651	0.007501
C	3.129318	−0.029540	−0.252501
C	−2.955049	0.937831	0.001444
C	2.693528	−1.289360	−0.227986
C	1.643027	0.272235	−0.173849
C	−1.905089	−0.624430	−0.042953
H	4.060046	0.508077	−0.302838
H	−4.324534	−0.858620	0.034319
H	−3.385061	1.930428	0.022938
H	3.124026	−2.281716	−0.250737
H	−0.306895	−1.540233	−0.103072
H	0.045670	1.187613	−0.091236

Table S18: NCN (ACR/DCR): formamidine dimer.

Atom	x	y	z
N	1.153084	1.445976	0.000000
N	−1.153084	−1.445976	0.000000
C	−0.030310	2.069030	0.000000
C	0.030310	−2.069030	0.000000
H	0.042857	3.162718	0.000000
H	−0.042857	−3.162718	0.000000
H	1.217250	0.414376	0.000000
H	−1.217250	−0.414376	0.000000
H	1.992112	1.992275	0.000000
H	−1.992112	−1.992275	0.000000
N	−1.157132	1.455729	0.000001
H	−1.923668	2.116104	0.000001
N	1.157132	−1.455729	0.000001
H	1.923669	−2.116104	0.000001

Table S19: NCO (DCR): formamide dimer.

Atom	x	y	z
O	−1.136110	1.439641	−0.000003
O	1.136109	−1.439640	−0.000002
N	1.135612	1.404248	0.000000
N	−1.135612	−1.404249	0.000001
C	−0.053463	2.005965	−0.000001
C	0.053463	−2.005964	0.000000
H	0.004662	3.108728	0.000001
H	−0.004660	−3.108727	0.000000
H	1.204837	0.378978	0.000000
H	−1.204837	−0.378979	0.000000
H	1.971773	1.959315	0.000002
H	−1.971774	−1.959316	0.000002

Table S20: NCO (ACR): formamide dimer.

Atom	x	y	z
H	−1.824531	−1.946515	0.101119
O	1.270573	−1.553617	−0.033180
H	0.072898	−3.147328	0.065503
N	−0.998905	−1.364986	0.050893
C	0.081056	−2.053400	0.030139
H	1.249090	−0.496659	−0.064227
O	−1.068055	1.208085	−0.024642
H	−1.045250	0.150878	0.008164
C	0.121127	1.708391	−0.091423
N	1.201364	1.020287	−0.111102
H	0.128044	2.802327	−0.129627
H	2.026447	1.602127	−0.165792

7 Other AZH (DCR) isomers

The current section reports the results computed for the two additional isomers of the AZH (DCR) dimer: bent−trans and planar.

7.1 Optimised geometries

All the geometries, given in Å, are reported as Cartesian coordinates.

Table S21: Planar isomer of the AZH (DCR) dimer.

Atom	x	y	z
O	1.159200	-1.699432	-0.000522
O	-1.132740	1.679418	-0.000220
N	1.196546	1.223577	0.000052
N	-1.170123	-1.243220	0.000225
C	0.021445	1.990371	0.000129
C	0.004722	-2.009316	-0.000259
C	0.862720	3.259907	0.000279
C	-0.836750	-3.279665	-0.000081
C	1.925625	2.440927	0.000149
C	-1.899541	-2.461071	0.000103
H	3.001577	2.539796	0.000068
H	-2.975548	-2.559129	0.000175
H	0.679417	4.318064	0.000229
H	-0.651735	-4.337516	-0.000064
H	-1.376613	-0.250055	-0.000011
H	1.402330	0.230283	-0.000254

Table S22: Bent−trans isomer of the AZH (DCR) dimer.

Atom	x	y	z
O	1.135706	1.592194	0.190670
O	-1.135709	-1.592197	-0.190659
N	1.665138	-0.725408	0.503582
N	-1.665136	0.725404	-0.503585
C	-3.274782	-0.350812	0.288966
C	3.274781	0.350816	-0.288965
C	-2.957771	0.935975	0.070268
C	2.957773	-0.935973	-0.070273
C	1.884711	0.665599	0.155250
C	-1.884713	-0.665601	-0.155245
H	4.144992	0.910779	-0.583466
H	-4.144996	-0.910771	0.583469
H	-3.441516	1.894968	0.196389
H	3.441520	-1.894964	-0.196400
H	0.840061	-1.187999	0.119945
H	-0.840059	1.187993	-0.119947

7.2 Energies and aromaticity metrics

Table S24 reports the electronic DFT energies of the different isomers of the AZH (DCR) dimers along with their aromaticity, as measured by the FLU and MCI indices.

Table S23: Electronic DFT energies (in Hartrees) along with the FLU and MCI aromaticity metrics for the different isomers of the AZH (ACR) dimer.

Isomer	E	FLU	MCI
Bent−trans	−491.721177	0.0636	0.0087
Bent−cis	−491.721935	0.0630	0.0088
planar	−491.710180	0.0724	0.0050

7.3 QTAIM and IQA values

The current section gathers the values of the QTAIM and IQA descriptors for the different isomers of the AZH (DCR) dimer. Atomic charges and delocalisation indices are given in electrons whereas all the energetic terms are reported in Hartrees.

Table S24: QTAIM atomic charges of the atoms involved in the HB contacts formed upon dimerisation.

Isomer	Q(D)	Q(H)	Q(A)	Q(C)
Bent−trans	−1.0612	0.4525	−1.2055	1.4179
Bent−cis	−1.0589	0.4540	−1.2051	1.4135
planar	−1.2077	0.5171	−1.2115	1.4901

Table S25: Delocalisation index (DI) along with the IQA interaction energy and its exchange-correlation and classical contributions for the D−H interaction.

Isomer	DI	E_{int}	E_{xc}	E_{cl}
Bent−trans	0.7114	−0.4206	−0.2250	−0.1956
Bent−cis	0.7078	−0.4207	−0.2241	−0.1966
planar	0.6410	−0.4655	−0.2058	−0.2597

Table S26: Delocalisation index (DI) along with the IQA interaction energy and its exchange-correlation and classical contributions for the H−A interaction.

Isomer	DI	E_{int}	E_{xc}	E_{cl}
Bent−trans	0.0635	−0.1393	−0.0134	−0.1259
Bent−cis	0.0655	−0.1413	−0.0138	−0.1275
planar	0.0742	−0.1705	−0.0162	−0.1543

Table S27: Delocalisation index (DI) along with the IQA interaction energy and its exchange-correlation and classical contributions for the D−C interaction.

Isomer	DI	E_{int}	E_{xc}	E_{cl}
Bent−trans	0.9103	−0.7811	−0.2851	−0.4960
Bent−cis	0.9101	−0.7767	−0.2848	−0.4918
planar	0.9454	−0.9404	−0.3008	−0.6396

Table S28: Delocalisation index (DI) along with the IQA interaction energy and its exchange-correlation and classical contributions for the C−A interaction.

Isomer	DI	E_{int}	E_{xc}	E_{cl}
Bent−trans	1.3063	−1.4706	−0.3992	−1.0714
Bent−cis	1.3060	−1.4682	−0.3990	−1.0692
planar	1.2812	−1.4914	−0.3966	−1.0948
